# Clinical observation on the efficacy of Tongdu Tuina manipulation in the treatment of primary enuresis in children

**DOI:** 10.1515/med-2023-0712

**Published:** 2023-06-29

**Authors:** Li-Pu Jie

**Affiliations:** Department of Paediatrics, The First People’s Hospital of Lanzhou, No. 1, Wujia West Street, Qilihe District, Lanzhou City, Gansu, 730050, China

**Keywords:** Tongdu Tuina, desmopressin acetate, children primary enuresis, clinical efficacy

## Abstract

The objective was to explore the efficacy of Tongdu Tuina manipulation in the treatment of primary single-symptom enuresis in children. A total of 102 children aged 5–16 with primary single-symptom enuresis were included in this study and randomly assigned to the Tuina group, the medication group and the control group, with 34 children in each group. The Tongdu Tuina group included manipulation of the Guanyuan, Qihai, Zhongji, Mingmen, kidney, Baihui, Sishencong and bladder acupoints, five times a week, the medication group was treated with 0.1 mg desmopressin acetate every night, and in the control group, the patients were given foods with high water content and underwent water deprivation 2 h before bedtime every night. The intervention time of each group was 1 month. The participants were followed up on Day 1 following treatment, as well as half a month, 1 month and 3 months after the implementation of the intervention measures, and the effective rate, the incidence of enuresis per week and the recurrence rate were calculated. As a result baseline demographic characteristics were comparable among 102 patients. Overall, 32 patients in the Tongdu Tuina group, 30 patients in the medication group and 34 patients in the control group completed the intervention. After half a month of treatment, there was no significant difference in the therapeutic efficacy among the three groups (*P* = 0.158), but each treatment could effectively reduce the frequency of weekly enuresis. The frequency of weekly enuresis in the Tongdu Tuina group was 3.8 ± 1.1 times, while that in the medication group was 4.0 ± 2.0 times. The frequency of weekly enuresis in the control group was 4.7 ± 1.8 times, and the difference was statistically significant (*P* = 0.016). After 1 month of treatment, the effective rates of the Tongdu Tuina group and the medication group were significantly increased (87.5% vs 83.33%, *P* < 0.0001), which was not the case with the control group. The frequency of enuresis was 1.9 ± 2.1 times per week in the Tongdu Tuina group, 2.4 ± 1.8 times per week in the medication group and 4.0 ± 0.9 times per week in the control group after 1 month of treatment. The difference between the three groups was statistically significant (*P* = 0.021), and there was a difference between the Tongdu Tuina group and the medication group (*P* < 0.0001). There was no significant difference between recurrence rate and the incidence of adverse events (*P* = 0.837, *P* = 0.856). In conclusion, both Tuina manipulation and desmopressin treatment can effectively improve children’s primary single-symptom enuresis with safety. However, Tongdu Tuina therapy may be superior to desmopressin treatment.

## 1 Introduction

Night enuresis, commonly known as urinary incontinence, is one of the most common developmental problems in childhood [1,2]. Enuresis refers to the occurrence of at least two instances of bed-wetting per week in children aged 5 years and above, with the issue lasting for at least 3 months [3–5]. Clinically, nocturnal enuresis is divided into primary and secondary nocturnal enuresis. Primary nocturnal enuresis is the most common [6,7], with the duration of this type at least 6 months [3,4]. In addition, nocturnal enuresis is also divided into single-symptom nocturnal enuresis and non-single-symptom nocturnal enuresis, with the former, also known as monosymptomatic nocturnal enuresis, having no daytime urinary tract manifestations, the only symptom being unconscious urination. However, the latter, non-monosymptomatic nocturnal enuresis, indicates that the patient has at least one additional lower urinary tract symptom, such as dysuria or diabetes insipidus [8,9].

According to statistics, around 16% of children aged five, 10% of children aged seven and 5% of children aged 11–12 have nocturnal enuresis, incidence rate is higher in males, while the gender ratio is around 1.5–2.1 [10]. While the natural recovery rate of nocturnal enuresis is around 15% per year with age, around 0.5–2% of children may continue to experience enuresis into adulthood without effective intervention [11,12]. Since enuresis has a significant impact on children’s self-esteem, sleep quality, quality of life, school performance and family life [13,14], adequate early intervention is crucial.

Desmopressin acetate is one of the first-line treatment methods [15]. This is a selective anti-diuretic hormone receptor type 2 agonist, which retains the anti-diuretic characteristics of anti-diuretic pressor activity. Previous studies have demonstrated that 60–70% of patients have improved nocturnal enuresis symptoms with this treatment, but recurrence following withdrawal is common [3,16]. Traditional Chinese medicine holds that the pathogenesis of enuresis in children is mostly deficiency-cold in the lower yuan and a weakness of the spleen and kidney, and is thus closely related to the kidney, bladder and spleen. The waist and abdomen points in the Du and Ren meridians have the functions of warming the kidney, consolidating the yuan and conditioning the bladder. Tuina massage can regulate the movement of the qi, blood and body fluid by dredging the Du and consolidating the Ren meridians, which has an impact on the visceral function and achieves a therapeutic effect, demonstrating a good curative effect in previous studies and considered to be suitable for promotion in primary medical institutions [17]. However, there exists no high-quality prospective study comparing Tuina and desmopressin acetate in the treatment of nocturnal enuresis. Therefore, this study provides a theoretical basis for the clinical application of Tongdu Tuina therapy by comparing the clinical efficacy, especially the long-term curative effect, of the two treatments.

## 2 Materials and methods

### 2.1 Research participants

Following a review of the relevant literature and an examination of the sample size of previous studies with good results [18], convenient sampling was used to select 134 patients with continuous primary single-symptom enuresis who stayed in The First People’s Hospital of Lanzhou City hospital from December 2017 to May 2018. The diagnostic criteria were determined with reference to the International Children’s Continence Society [19]. The inclusion criteria were as follows: (1) 5–16 years of age, (2) the incidence of enuresis was >3 times a week for more than 6 months, (3) independent daytime control of urination but no independent control during sleep, (4) patients who were conscious, with no mental illness, who (or their parents) agreed with the treatment and signed the informed consent form, (5) patients with no serious diseases of the heart, liver, kidney, hematopoietic system and endocrine system that may affect functional recovery and (6) patients with no organic diseases of the urinary system, urinary tract infection, diabetes insipidus, neurogenic bladder or urinary tract malformation. The exclusion criteria were as follows: (1) patients taking drugs for enuresis 14 days before the start of the study, (2) patients who met the contraindications of desmopressin acetate, (3) patients with serious heart diseases, haemorrhagic diseases or other diseases that are not suitable for Tuina treatment.

In the review stage of the study population, a detailed clinical examination was performed on the patients, and the parents were interviewed to obtain a detailed disease history of the patients. According to the clinical examination results and the disease history, 32 patients did not meet the inclusion criteria, including three who were aged >16 years, nine aged <5 years, six with fewer than three incidences of enuresis per week, seven whose parents did not agree to participate in the treatment, three with epilepsy, three with a urinary tract infection and one with a urinary system organic disease. A final total of 102 patients met the inclusion criteria and were numbered according to the admission time. The participants were then divided into the Tuina group, the oral desmopressin acetate group (medication group) and the control group using a random number table. This study received ethical approval from the hospital.

### 2.2 Intervention methods

In the Tongdu Tuina group, the patients were given foods with high water content and underwent water deprivation 2 h before bedtime every night. The main acupoints of Tuina are Guanyuan, Qihai, Zhongji, Mingmen, Shenshu, Baliao and chiropractic, while the accompanying acupoints are Baihui, Sishencong, bladder acupoints, large intestine acupoints, Jiaji, kidney top, Guiwei, Laogong, Waiguan, Zusanli, Sanyinjiao and Yanglingquan. In general, 3–4 acupoints are selected according to the specific situation. First, the manipulation was relaxed for a moment before the acupoints were pushed using four fingers with light, soft and deep penetration. Finally, the acupoints passed by Rendu were wiped by the palm for heating. This treatment was administered once a day, five times a week for 4 weeks.

The medication group, the patients were given foods with high water content and underwent water deprivation 2 h before bedtime every night. Half an hour before bedtime, 0.1 mg of desmopressin (DDAVP; Heyue, Hainan Zhonghe Pharmaceutical Co., Ltd) was swallowed with a small amount of water. After the medication, water intake was forbidden until the next morning, once every night, for 1 month. If adverse reactions occur during treatment, a timely examination is required and if abnormal results are returned, the drug treatment must be stopped immediately.

Control group: The patients were given foods with high water content and underwent water deprivation 2 h before bedtime every night.

### 2.3 Follow-up and observation indexes

Follow-ups were performed at baseline (Day 1 after treatment, t1), as well as half a month (15 days, t2), 1 month (30 days, t3) and 3 months (90 days, t4) after the end of one course of treatment. The follow-up indicators included effective number, weekly enuresis frequency and recurrence rate. The clinical efficacy was evaluated as follows: (1) cure: the enuresis symptoms had disappeared, (2) effectual: the incidence of enuresis decreased by >50%, (3) effective: the incidence of enuresis decreased by <50%, and (4) invalid: no decrease in the incidence of enuresis. The incidence of enuresis was obtained according to the daily enuresis logs recorded by the patient’s parents.

### 2.4 Statistical analysis

The data were analysed using SPSS26.0 software. The quantitative data were expressed in terms of mean and standard deviation ( \bar{X}\pm S] ), and the qualitative data were expressed in terms of the adoption rate or composition ratio (%). A *t*-test was used to compare the two groups of quantitative data, variance analysis was used to compare the two groups of quantitative data and a Student–Newman–Keuls test was used to compare them. A chi-square test was used to compare the qualitative data, with the rank-sum test used if it was a rank variable; the test level was *α* = 0.05.


**Ethics approval and consent to participate:** This study was conducted in accordance with the declaration of Helsinki. This study was conducted with approval from the Ethics Committee of The First People’s Hospital of Lanzhou City. Written informed consent was obtained from all participants.

## 3 Results

### 3.1 Basic characteristics of study population

**Figure 1:**
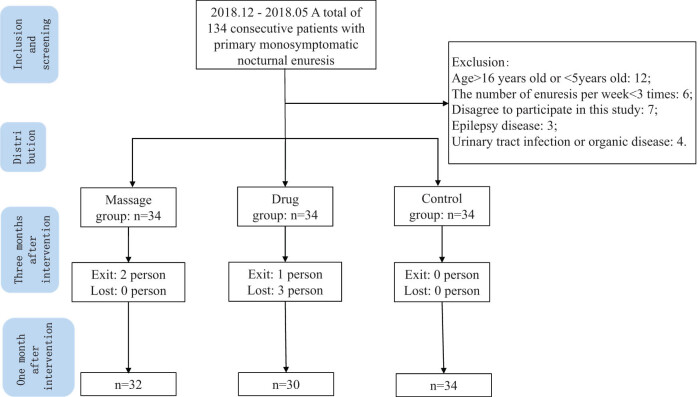
Screening, allocation and follow-up of study population.

After screening, 102 patients met the inclusion and exclusion criteria of this study. After half a month of treatment (t2), two patients in the Tuina group withdrew from the study, while one patient in the medication group withdrew and three could not complete the follow-up. The other study participants completed both the intervention and the follow-up. Figure 1 shows the screening, distribution and follow-up processes used, while Table 1 presents the basic demographic characteristics of the study population. The study population was concentrated in the 8–9 age group, and the main caregiver was the mother. The incidence of enuresis per week was 5.2 ± 0.9 in the Tuina group, 6.1 ± 2.1 in the medication group and 5.8 ± 1.2 in the control group. The basic characteristics of the three groups were comparable, with the differences not statistically significant (*P* = 0.652, *P* = 0.599, *P* = 0.212, *P* = 0.195).

**Table 1:** Basic characteristics table of research object [ \bar{X}\pm S] ; *n* (%)]

Group	*N*	Age (years)	Male (%)	Weekly enuresis times	Caregiver (father/mother)
Tuina group	34	8.7 ± 2.1	15	5.2 ± 0.9	8/26
Drug group	34	8.5 ± 1.1	14	6.1 ± 2.1	5/29
Control group	34	8.1 ± 1.9	18	5.8 ± 1.2	4/30
*F*/*χ* ^2^		1.213	1.026	2.131	0.399
*P*		0.652	0.599	0.212	0.195

### 3.2 Treatment efficiency

Half a month after the treatment (t2), the therapeutic effect across the three groups was not significant, with the differences also not statistically significant (*P* = 0.158). However, 1 month after the treatment (t3), the therapeutic effect of the three groups was significantly improved. The cure rate of the Tuina group was 46.88%, and the total effective rate was 87.50%, while the cure rate of the medication group was 40.00%, with the total effective rate 83.33%, and the cure rate of the control group was 0%, with the total effective rate 14.71%. Given that the number of cases of cure, effectual or effective was relatively small, a trend chi-square test was not performed, with a chi-square test only used to evaluate the total efficiency of the three groups. While the difference in the effective rate across the three groups was statistically significant (*P* < 0.0001), the pairwise comparative analysis indicated that there was no significant difference in effective rate between the Tuina group and the medication group. However, compared to the control group, the enuresis was improved in the Tuina group and the medication group (*P* < 0.0001) (Table 2).

**Table 2:** Comparison of curative effect of three groups after half month and 1 month of treatment [*n* (%)]

Group	*N*	Cure	Effectual	Effective	Invalid	Total effective rates
**Treatment for half a month**						
Tuina group	32	3(9.37)	1(3.13)	3(9.37)	25(78.13)	7(21.88)
Drug group	30	1(3.33)	2(6.67)	1(3.33)	26(86.67)	4(13.33)
Control group	34	0(0.00)	1(2.94)	1(2.94)	32(94.12)	2(5.88)
*χ* ^2^						3.696
*P*						0.158
**Treatment for 1 month**						
Tuina group	32	15(46.88)	6(18.75)	7(21.87)	4(12.50)	28(87.50)^a^
Drug group	30	12(40.00)	7(23.33)	6(20.00)	5(16.67)	25(83.33)^a^
Control group	34	0(0.00)	2(5.88)	3(8.83)	29(85.29)	5(14.71)
*χ* ^2^						43.677
*P*						<0.0001

Note: ^a^The difference was statistically significant compared with the control group, *P* < 0.0001.

### 3.3 Frequency of enuresis

The weekly enuresis frequency was significantly increased both after 1 month and after half a month of treatment. Half a month after the treatment (t2), the frequency of enuresis was 3.8 ± 1.1 times per week in the Tuina group, 4.0 ± 2.0 times per week in the medication group and 4.7 ± 1.8 times per week in the control group. The difference across the three groups was statistically significant (*P* = 0.016). One month after the treatment, the frequency of enuresis was 1.9 ± 2.1 times per week in the Tuina group, 2.4 ± 1.8 times per week in the medication group and 4.0 ± 0.9 times per week in the control group, with the difference across the three groups statistically significant (*P* = 0.021). Compared to in the control group, the frequency of enuresis in the medication and Tuina groups was significantly improved after half a month and after 1 month of treatment, and the difference was statistically significant (*P* < 0.0001). The frequency of enuresis in the Tuina group was significantly lower than that in the medication group, and the difference was statistically significant (*P* < 0.0001) (Table 3).

**Table 3:** Comparison of weekly enuresis frequency among three groups after half and 1 month of treatment

Group	Baseline	Treatment for half a month	Treatment for 1 month
Tuina group	5.2 ± 0.9	3.8 ± 1.1^a^	1.9 ± 2.1^ab^
Drug group	6.1 ± 2.1	4.0 ± 2.0^a^	2.4 ± 1.8^a^
Control group	5.8 ± 1.2	4.7 ± 1.8	4.0 ± 0.9
*F*	2.131	3.638	3.002
*P*	0.212	0.016	0.021

Note: ^a^The difference was statistically significant compared with the control group, *P* < 0.0001. ^b^The difference was statistically significant compared with the drug group, *P* < 0.0001.

### 3.4 Recurrence rate

Three months after treatment (t4), the cured patients were followed up. One patient (6.67%) in the Tuina group and two (16.67%) in the medication group had relapsed. However, the difference between the two groups was not statistically significant (*P* = 0.837).

### 3.5 Adverse events

One case of hyponatremia occurred in the drug group during treatment, but the patient returned to normal after a limited intake of drinking water. There were two cases of eczema caused by enuresis in the Tuina group and the medication group, and three such cases in the control group. There was no significant difference in the incidence of adverse events among the three groups (*P* = 0.856).

## 4 Discussion

This study evaluated the clinical efficacy and safety of Tongdu Tuina manipulation and desmopressin acetate in the treatment of primary single-symptom enuresis. The results indicated that 21.88% of the patients in the Tuina group were improved after half a month of treatment, with the frequency of enuresis down to 3.8 ± 1.1 times per week. The effective rate after 1 month of treatment was 87.50%, with the frequency of enuresis decreasing to 1.9 ± 2.1 times per week. The effective rate was 13.33% and the frequency of enuresis was 4.0 ± 2.0 times a week after taking desmopressin acetate for half a month. One month after the medication treatment, the effective rate was 83.33% and the frequency of enuresis decreased to 2.4 ± 1.8 times a week. Three months after the treatment, the recurrence rate of the Tuina group was not statistically significant compared to that of the medication group, being 10% lower in the former, indicating a certain clinical significance.

At present, it is widely recognized that nocturnal enuresis is related to specific psychological and genetic factors, as well as neurogenesis and bladder dysfunction in children [20]. According to the theory of traditional Chinese medicine, enuresis is related to congenital defects, but the aetiology of children’s enuresis has not yet been fully elucidated. However, it can be clarified that children’s enuresis is related to deficiency-cold of the lower Yuan, deficiency of the kidney qi and damp-heat of the liver meridian [21]. Western medicine therapy mainly includes drug intervention and awakening therapy. However, previous studies have demonstrated that while wake therapy has a positive effect on the treatment of enuresis, it may repeatedly interrupt a child’s sleep, thereby reducing both sleep quality and compliance [22–24]. Meanwhile, although desmopressin acetate is a first-line treatment, adverse events, such as hyponatremia, may still occur during long-term treatment [25].

Tuina manipulation has unique advantages in the treatment of nocturnal enuresis in children. A systematic evaluation of 12 randomized controlled trials indicates that the therapeutic effect of Tuina manipulation is more obvious than that of traditional Chinese medicine (risk ratio = 1.45; 95% confidence interval: 1.31, 1.61) [18]. Tuina therapy using specific acupoints (Baihui, Qihai, Zhongji, Mingmen, kidney acupoints, etc.) balances the Yin and Yang and plays a role in tonifying any deficiency, the operation of the body’s qi and blood and the visceral function activities, achieve the effect of treating infantile enuresis.

## 5 Limitations

This study has certain limitations. First, the study was based on a single centre and involved a comparatively small sample, meaning some bias could have been introduced and the research population may lack certain representativeness. Multi-centre data are needed to improve the credibility of the results. In addition, the study population consisted of children with good intellectual development, and the effect of Tongdu Tuina therapy and desmopressin acetate on children with poor intellectual development could not be evaluated. Finally, due to the limitation of funding in this study, only the clinical effect of 1 month of intervention was explored, meaning the effect and safety of long-term intervention could not be investigated.

## 6 Conclusion

In summary, Tongdu Tuina and desmopressin acetate are effective and successful treatment options for primary single-symptom enuresis in children, with both treatments effectively improving the cure rate and reducing the weekly frequency. Compared to desmopressin acetate treatment, the recurrence rate and the incidence of enuresis were clearly improved following Tongdu Tuina therapy, which provides an effective treatment option for the treatment of enuresis in children and is worthy of universal promotion in clinical practice.
